# Supplementary material for: Three-dimensional context rather than NLS amino acid sequence determines importin α subtype specificity for RCC1
Source: Nat Commun. 2017 Oct 17;8:979. doi: 10.1038/s41467-017-01057-7 (PMC5645467; doi:10.1038/s41467-017-01057-7)
Supplement: Supplementary file 1 — Supplementary Information [file 41467_2017_1057_MOESM1_ESM.pdf]

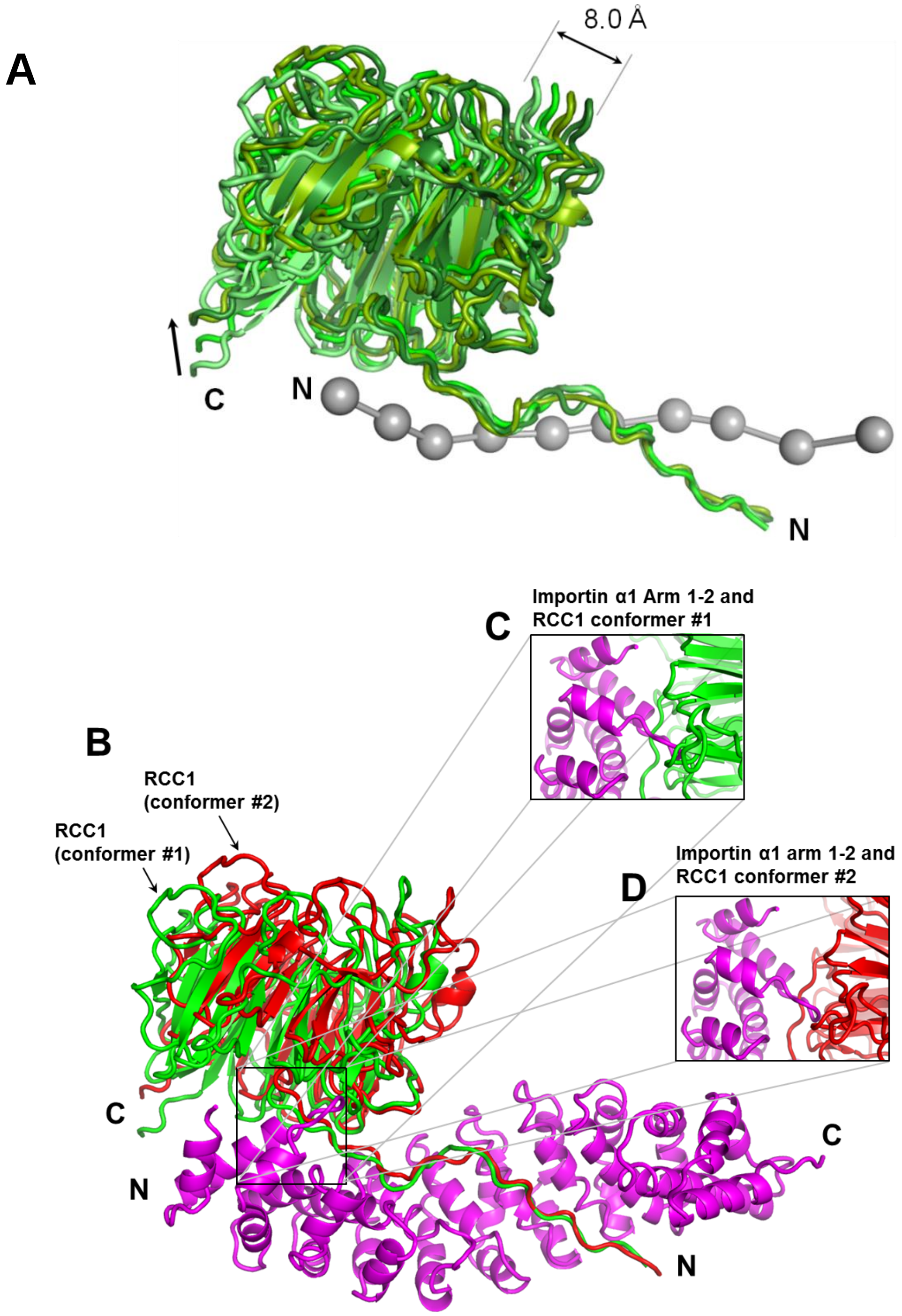

Supplementary Figure 1: (A) Superimposition of the eight importin α3:RCC1 complexes in the triclinic asymmetric unit. Importin α3 is nearly identical in the eight complexes (RMSD ~1.5 Å) and is shown as beads-on-a string (e.g. each bead represents the position on one Armadillo repeat), while four of the eight RCC1 are shown as ribbons. Global superimposition (in Coot [1]) of the eight RCC1 protomers observed crystallographically reveals a maximum displacement of ~8 Å. (**B**) **Superimposition of the two most distant RCC1 conformers (colored in green and red) docked onto the structure of importin α1 (colored in magenta).** The zoom-in panels in **C** and **D** show regions of clashing between RCC1 and importin α1.

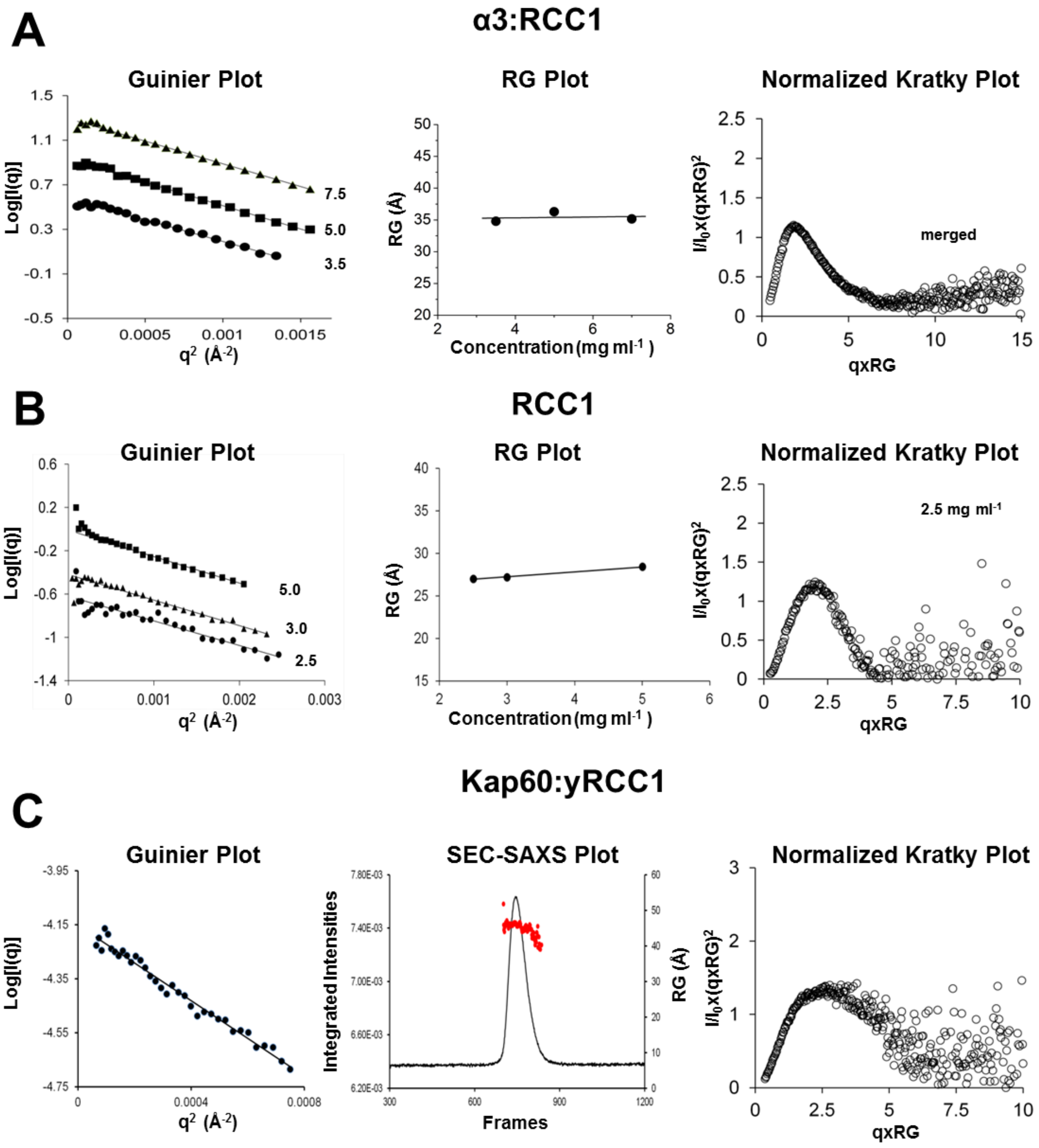

Supplementary Figure 2: Quality of SAXS data measured using (A-B) conventional SAXS (α3:RCC1 and human RCC1) and (C) SEC-SAXS (Kap60:yRCC1complex). The *left* panels show Guinier plots calculated at different protein concentrations, except in (**C**) that shows SEC-SAXS data. The *middle* panels in (**A**) and (**B**) are plots of RG versus concentration. In (**C**) the middle panel displays integrated intensities and RG (on y-axis) against number of frames on the X-axis (the red dots indicate RG values corresponding to frames on X-axis). The *right* panels show normalized Kratky plots calculated from SAXS data. All SAXS data were analyzed using ATSAS data analysis software [2].

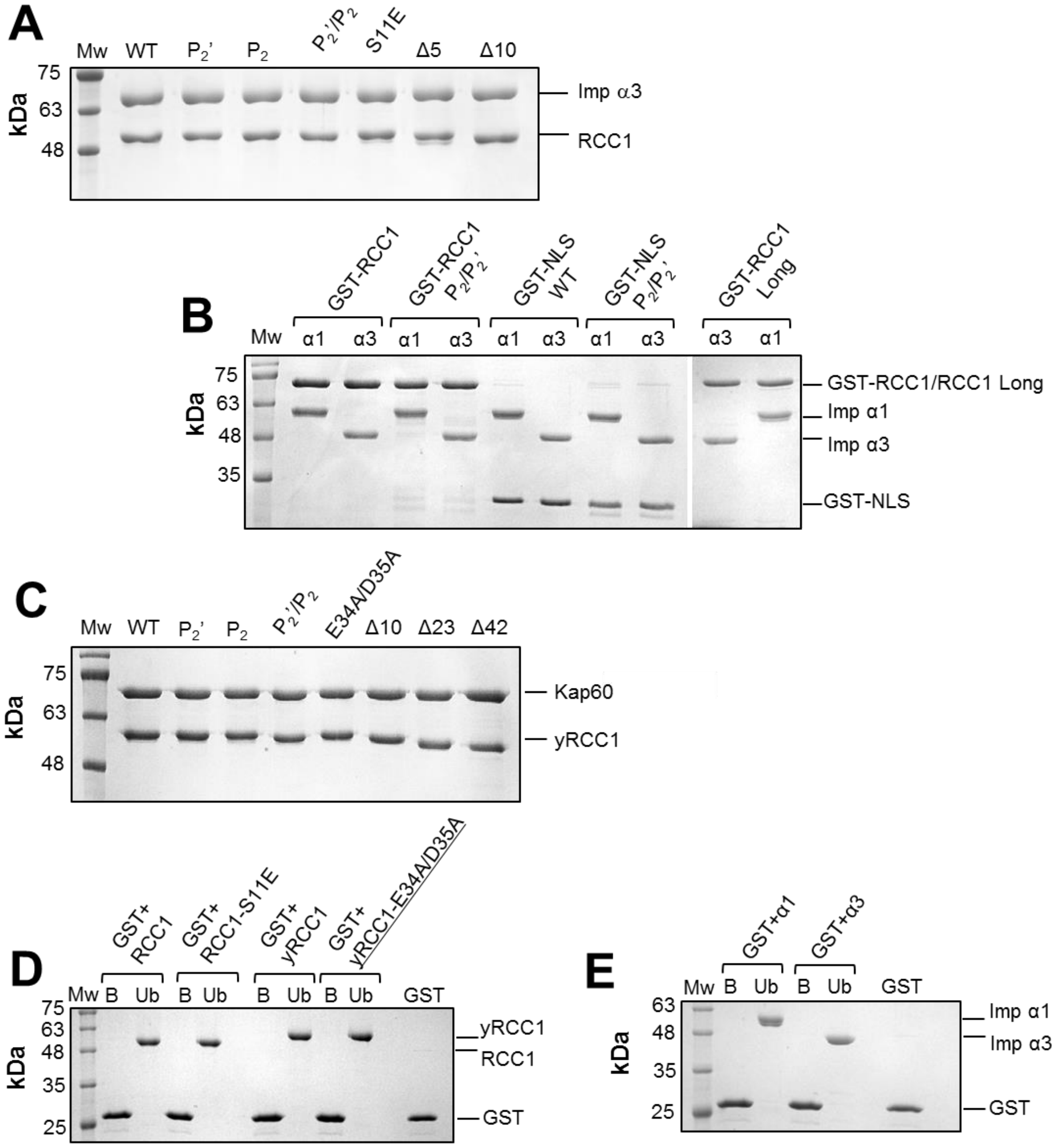

Supplementary Figure 3: Loading controls for pull-down experiments in Fig. 3b (**A**)**, 4d** (**B**) **and 7d** (**C**)**.** (**A**) GST-ΔIBB-importin α3 (1 μM) was used to pull down His-RCC1 or RCC1-mutants (0.75 μM); (**B**) GST-RCC1/RCC1-Long or RCC1-NLS (0.75 μM) was used to pull down ΔIBB-importin α3 or ΔIBB-importin α1 (1 μM); (**C**) GST-ΔIBB-Kap60 (1 μM) was used to pull down His-yRCC1 or its mutants (0.75 μM). (**D,E**) Control pull-downs showing that free GST does not associate with free RCC1 or importin α3/Kap60. (**D**) Free GST (1 μM) was used to pull down His-RCC1 or its mutants (0.75 μM). (**E**) Free GST (1 μM) was used to pull down ΔIBB-importin α1/3 (1 μM). The abbreviations in panels **D** and **E** are: 'B'=bound species and 'Ub'=unbound species.

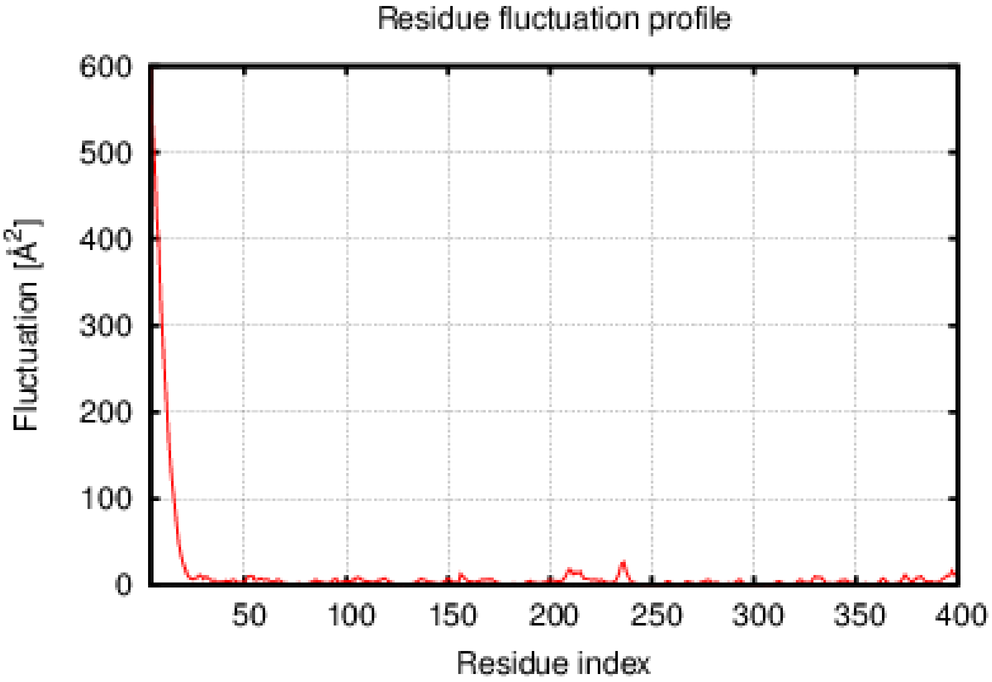

Supplementary Figure 4: RCC1 N-terminal tail is highly flexible. Plot of the Root Mean Square Fluctuations (RMSF or simply 'Fluctuation') of RCC1 backbone Cα atoms (Y-axis) observed during (MD) simulations. RCC1 N-terminal tail (res. 1-30) has the greatest RMSF. The simulation was run using the extended conformation of RCC1 observed in the crystal structure as starting model. MD simulations were performed using CABS-flex server (http://biocomp.chem.uw.edu.pl/CABSflex) for fast simulation of protein structure fluctuations [3].

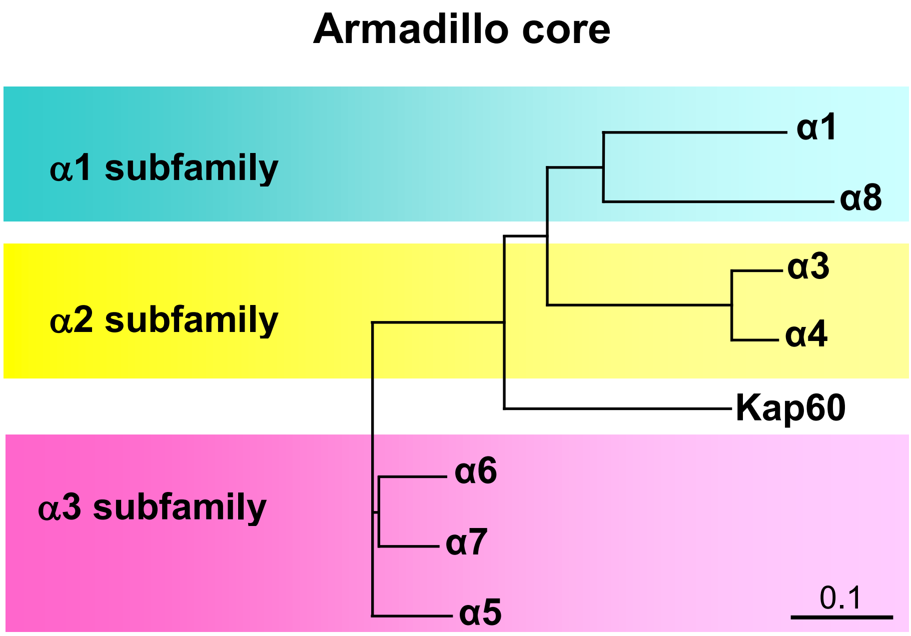

Supplementary Figure 5: Phylogenetic tree of human importin α isoforms and Kap60. Phylogenetic tree for human importin α isoforms and yeast Kap60 generated using Clustal Omega [4] and Tree View [5]. Only the amino acid sequence of the Armadillo core was used in this alignment. Kap60 is more similar to human isoforms of the α2 subfamily than importin α1.

A

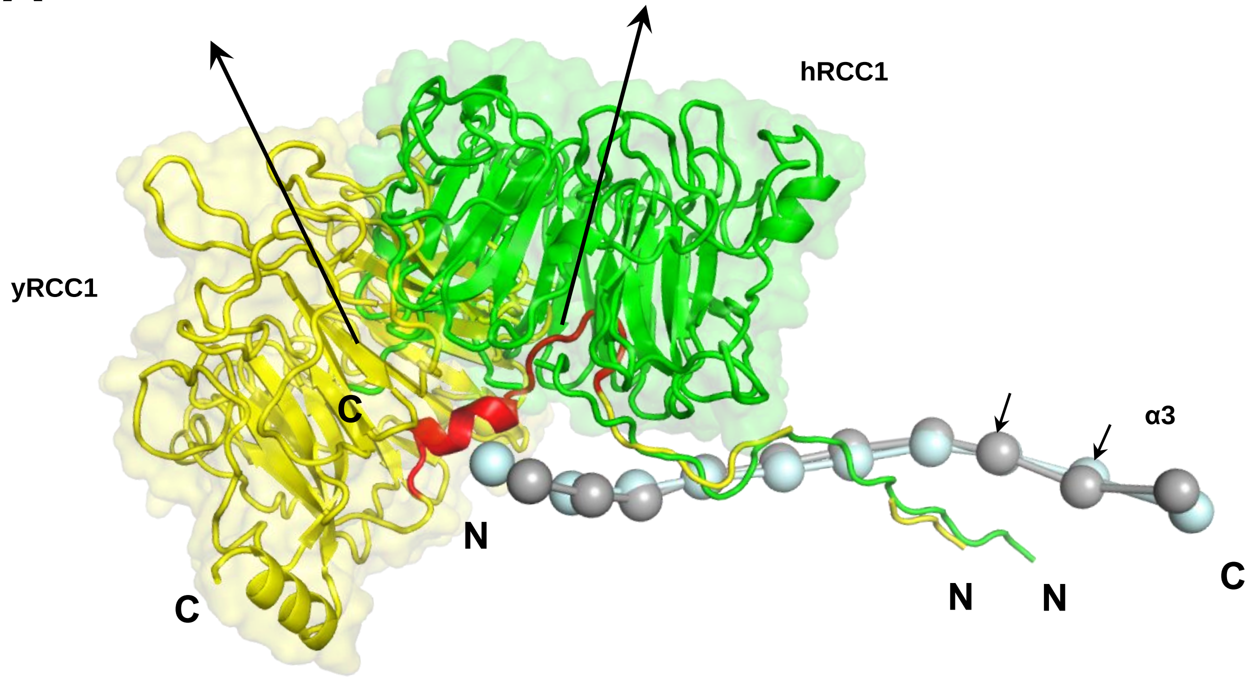

B

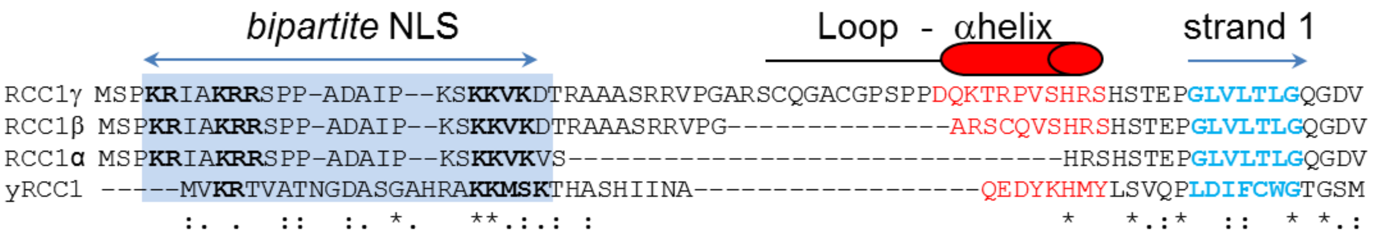

Supplementary Figure 6: (**A**) **Structural alignment of human importin α3:RCC1 and yeast Kap60:yRCC1 complexes**, with importin α3 (gray) and Kap60 (cyan) shown as beads-on-a-string and human/yeast RCC1 colored in green and yellow, respectively. The arrows around the center of mass of the RCC1 β-propellers illustrate the very different position of this domain in the two crystallographic complexes. The loop-helix moiety of yRCC1 is colored in red. (**B**) **Sequence alignment of the first 60 residues of human RCC1 isoforms and yeast RCC1.** A putative 'loop-helix' motif (found in yRCC1 but missing in RCC1α) also exists in human isoforms β and γ. Sequence alignment was performed using Clustal Omega [4].

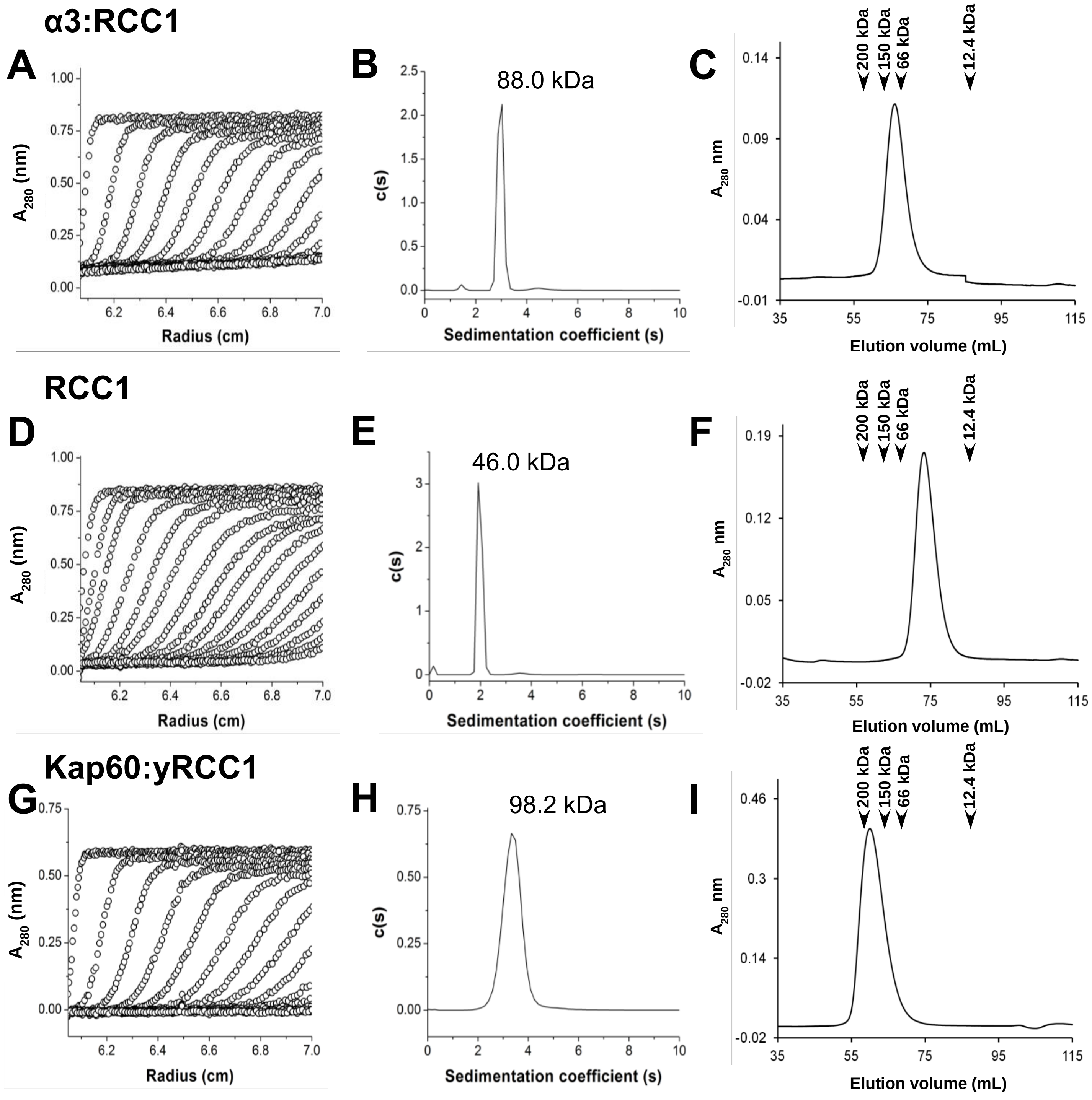

Supplementary Figure 7: Analytical Ultracentrifugation Sedimentation Velocity (AUC-SV) and Size Exclusion Chromatography (SEC) reveal all samples used for SAXS analysis are monodisperse. Panels (**A**), (**D**) and (**G**) show the position of the sedimentation boundary during AUC-SV analysis for the importin α3:RCC1 complex, free RCC1 and

Kap60:yRCC1 complex, respectively, at ~0.9 mg ml^{-1}. In all three cases, the sedimentation boundary exhibits monophasic behavior, which is indicative of a single major component in solution, migrating with sedimentation coefficients of 2.97S (**B**), 1.98S (**E**) and 3.37S (**H**). Conversion of the distribution of the apparent sedimentation coefficient to molecular mass for three independent runs revealed a M.W. of ~88.0 ± 2 kDa (importin α3:RCC1 complex), ~46.0 ± 1.7 kDa (free RCC1) and ~98.2 ± 2.3 kDa (Kap60:yRCC1 complex) (**Supplementary Table 1**). The oligomeric state observed by AUC-SV was concentration-independent under the range of concentrations tested (between 1-5 mg ml^{-1}). (**C, F, I**) SEC analysis of purified importin α3:RCC1 complex, free RCC1 and Kap60:yRCC1 analyzed on a Superose 12 gel filtration column calibrated with M.W. markers (whose elution volumes and relative M.W. are indicated by arrows). The approximate masses obtained from M.W. markers are: ~103 kDa and 49 kDa for the α3:RCC1 complex and free RCC1, respectively, while the Kap60:yRCC1 complex migrates like a ~177 kDa species, possibly consistent with an elongated 1:1 monomer (**Fig. 6a**).

Figure 3b

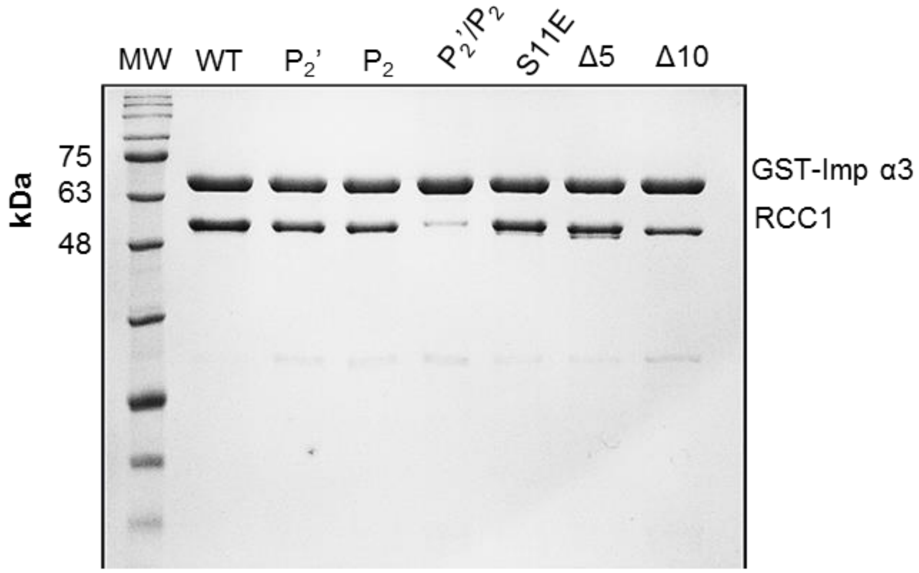

Figure 7d

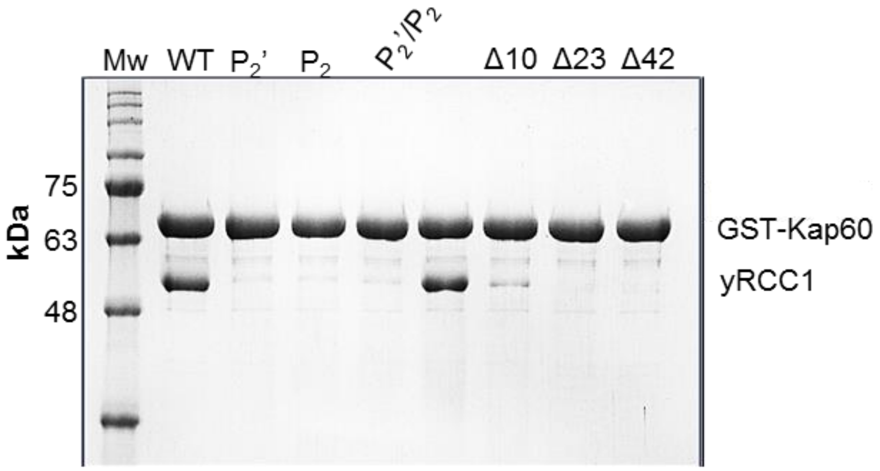

Supplementary Figure 8: Uncropped gels in Fig 3b and 7d

Supplementary Table 1. Biophysical parameters measured by SAXS and AUC

Species	α3:RCC1	Human RCC1	Kap60:yRCC1
**SAXS**			
**Data collection and structural parameters**			
Instrument	BioSAXS-2000	BioSAXS-2000	G1-beamline
Wavelength (Å)	1.56	1.56	0.999
Exposure time (s)	300	300	1
Protein Concentration (mg ml^{-1})	3.5-7.5	2.5-5.0	0.01-0.5
Temperature (K)	283	283	277
Radius of Gyration, RG * / RG from crystal structure (Å)	34.6 ± 0.9 / 32.7	27.8 ± 0.7 / 23.3	45.9 ± 0.5 / 39.0
Maximum Diameter, D_{max} * / Max dimension crystal structure (Å)	115.3 / 110.5	102.5 / 98.6	137.0 / 135.0
Porod M.W.** / Theoretical M.W. (kDa)	98.5 / 91.2	55.9 / 49.0	99.0 / 103.0
**Software employed**			
Primary Data Reduction	Rigaku ATSAS pipeline	Rigaku ATSAS pipeline	RAW
Data Processing	ATSAS	ATSAS	ATSAS
Ab initio analysis	DAMMIF	DAMMIF	DAMMIF
Validation and averaging	DAMAVER	DAMAVER	DAMAVER
Rigid-body modelling	SITUS	SITUS	SITUS
Computation of model intensities	FoxS	FoxS	FoxS
3D-graphics representations	PyMOL	PyMOL	PyMOL
**AUC-SV**			
Apparent Sedimentation Coef., s (S)	2.97	1.98	3.37
Absolute Sedimentation Coef., $s_{20,w}$ (S)	4.85	2.73	5.36
Estimated / Expected M.W. (kDa)	88.0 / 91.2	46.0 / 49.0	98.2 / 103.0
Frictional Ratio, f/f_0	1.45	1.7	1.42
Radius of Hydration, RH (Å)	42.7	40.6	43.4

* Reported for merged data (3.5, 5 and 7.5 mg ml^{-1}) for α3:RCC1 and data at 2.5 mg ml^{-1} for RCC1. For Kap60:yRCC1, SEC-SAXS was carried out from a sample injected at 5 mg ml^{-1} onto a Superose 12 column.
** Porod M.W. is calculated as the Porod volume (A^3) / 1.8.

Supplementary Table 2. Structural alignment of RCC1-NLS with representative NLSs solved in complex with human, yeast and rice importin α

NLS type	Minor Binding Site $P_{-1}'P_0'P_1'P_2'P_3'P_4'P_5'$		Major Binding Site P_{-1} P_0 P_1 P_2 P_3 P_4 P_5	PDB id
**SV40T-ag**	P K K **K** R K		P K **K** K R K V	1EJL/1BK6*
**hPLSCR1-NLS**			G **K** I S K HWTGI	1Y2A
**hPLSCR4-NLS**	S I I **R** K W N			3Q5U
**Guα-NLS**	G Q K **R** S F S			3ZIN
**A89-NLS**	L G K **R** K Y W			4B8P**
**B54-NLS**	L G K **R** K R H			2YNS**
**TPX2**	K **R** K H		P V **K** M I K	3KND
**C-Myc**	K **R** V K L		P A A **K** R V K	1EE4*
**NP**	A V K **R** P A A	TKKAG	Q A **K** K K K L D	1EJY/1EE5*
**Kap60-IBB**	E L R **R** R R D	TQQVELRKAKRDEA	L A **K** R R N F	1WA5*
**h1NLS**	TR K K **R** K D P	DSDDWSES	N S **K** E N K ID	4XZR *
**h2NLS**	T N K **R** K R E	QISTDNEAKMQIQEEKS	P K **K** K R K KRSSKANK	4PVZ*
**hRCC1**	KRI A K **R** R S	PPADAIP	K S **K** K V K V	5TBK
**yRCC1**	M V K **R** T V A	TNGDASGAH	R A K **K** M S K TH	5T94*

* denotes yeast importin α (Kap60).
** denotes rice importin α. In all other cases, mammalian importin α was co-crystallized with NLSs

Supplementary Table 3. Nucleotide sequence of synthetic primers and the RCC1 gene used in this study

**Primers used for human RCC1**	
RCC1_R9A-for	AAGCGTATTGCCAAAGCGCGCAGTCCTCCGGCC
RCC1_R9A-rev	GGCCGGAGGACTGCGCGCTTTGGCAATACGCTT
RCC1_K21A-for	GCCATCCCGAAAAGCGCGAAAGTGAAGGTTAGC
RCC1_K21A-rev	GCTAACCTTCACTTTCGCGCTTTTCGGGATGGC
RCC1_S11E-for	ATTGCCAAACGTCGCGAACCTCCGGCCGATGCC
RCC1_S11E-rev	GGCATCGGCCGGAGGTTCGCGACGTTTGGCAAT
RCC1_Del5-for	CAGGGGCCCGGATCCATTGCCAAACGTCGC
RCC1_Del5-rev	GCGACGTTTGGCAATGGATCCGGGCCCCTG
RCC1_ Del10-for	TTCCAGGGGCCCGGATCCAGTCCTCCGGCCGATGCC
RCC1_ Del10-rev	GGCATCGGCCGGAGGACTGGATCCGGGCCCCTGGAA
RCC1 NLS_ 1-27-for	CGCTGGATCCATGAGCCCGAA
RCC1 NLS_1-27-rev	AGCGCTCGAGTTAGTGGCTAACCTTCACTTT
RCC1-long-for	AAGAAAGTGAAGGTTAGCGGTTCCGCAGGTTCTGCCGCGGGATCTGGCG AATTCCACCGCAGCCATAGCACC
RCC1-long-rev	GGTGCTATGGCTGCGGTGGAATTCGCCAGATCCCGCGGCAGAACCTGCG GAACCGCTAACCTTCACTTTCTT
**Primers used for human yeast RCC1**	
yRCC1-R4A-f	GAATTCATGGTCAAAGCAACAGTCGCCACCAAT
yRCC1-R4A-r	ATTGGTGGCGACTGTTGCTTTGACCATGAATTC
yRCC1-K20A-f	GCTCATAGAGCAAAGGCAATGTCTAAAACTCAT
yRCC1-K20A-r	ATGAGTTTTAGACATTGCCTTTGCTCTATGAGC
yRCC1-E34A/D35A-f	ATCATAAACGCTCAAGCAGCATACAAGCATATGTAC
yRCC1-E34A/D35A-r	GTACATATGCTTGTATGCTGCTTGAGCGTTTATGAT
yRCC1-del10-f	CACGGAATTCGACGCGTCTGGCGCTCA
yRCC1-del10-r	CACGCTCGAGTTAATCATCCATTTC
yRCC1-del23-f	CACGGAATTCACTCATGCTTCTCATATCATAA
yRCC1-del23-r	CACGCTCGAGTTAATCATCCATTTC
yRCC1-del42-f	CACGGAATTCGTCCAACCATTGGATATATTTT
yRCC1-del42-r	CACGCTCGAGTTAATCATCCATTTC
**Human RCC1 synthetic gene**	
ATGAGCCCGAAGCGTATTGCCAAACGTCGCAGTCCTCCGGCCGATGCCATCCCGAAAAGCAAGAAAG<br>TGAAGGTTAGCCACCGCAGCCATAGCACCGAACCGGGTCTGGTTCTGACCCTGGGCCAAGGCGATG<br>TTGGCCAGCTGGGTCTGGGTGAGAACGTGATGGAGCGCAAAAAACCGGCCCTGGTTAGCATTCCGG<br>AAGATGTTGTGCAGGCCGAAGCAGGCGGCATGCATACCGTTTGTCTGAGCAAAAGCGGCCAGGTGTA<br>CAGCTTTGGCTGTAACGACGAAGGTGCCCTGGGTCGTGACACAAGCGTGGAAGGCAGTGAAATGGT<br>GCCGGGTAAAGTGGAACTGCAGGAGAAGGTGGTGCAGGTGAGCGCAGGCGATAGCCATACCGCCGC<br>CTTAACCGACGATGGTCGCGTTTTTCTGTGGGGCAGCTTCCGCGATAACAACGGCGTGATCGGTCTG<br>CTGGAACCGATGAAGAAAAGCATGGTGCCGGTGCAAGTGCAGCTGGATGTTCCGGTGGTGAAAGTTG<br>CAAGCGGTAACGACCACCTGGTTATGCTGACAGCCGATGGCGATCTGTATACACTGGGCTGTGGTGA<br>ACAGGGCCAATTAGGTCGTGTTCCGGAACTGTTCGCAAATCGTGGTGGTCGCCAGGGCCTGGAACGT<br>CTGCTGGTGCCTAAATGCGTGATGCTGAAAAGCCGTGGTAGCCGTGGCCACGTGCGCTTTCAGGATG<br>CCTTTTGCGGCGCCTACTTTACCTTCGCCATTAGCCACGAGGGCCACGTTTACGGCTTTGGCCTGAG<br>CAACTATCACCAGCTGGGTACCCCGGGCACCGAAAGCTGCTTCATCCCGCAAAACCTGACCAGCTTC<br>AAGAACAGCACAAAGAGCTGGGTGGGCTTCAGCGGTGGCCAGCACCATACCGTGTGCATGGATAGC<br>GAAGGTAAAGCCTACAGCCTGGGTCGCGCAGAATATGGTCGCTTAGGTCTGGGCGAAGGCGCCGAA<br>GAGAAAAGCATTCCGACACTGATTAGCCGCCTGCCGGCAGTTAGCAGCGTTGCATGCGGTGCAAGCG<br>TGGGTTATGCCGTTACCAAAGATGGCCGCGTGTTTGCATGGGGCATGGGTACCAATTACCAGCTGGG<br>CACCGGTCAGGATGAGGATGCATGGAGCCCTGTGGAAATGATGGGCAAACAGCTGGAGAACCGCGT<br>TGTGCTGAGTGTGAGCAGCGGCGGCCAGCACACCGTTTTACTGGTGAAGGACAAGGAGCAGAGTTA<br>G	

SUPPLEMENTARY REFERENCES

1. Emsley, P. & Cowtan, K. Coot: model-building tools for molecular graphics. *Acta Crystallogr D Biol Crystallogr* **60**, 2126-32 (2004).

2. Petoukhov, M.V. et al. New developments in the ATSAS program package for small-angle scattering data analysis. *J Appl Crystallogr* **45**, 342-350 (2012).

3. Jamroz, M., Kolinski, A. & Kmiecik, S. CABS-flex: Server for fast simulation of protein structure fluctuations. *Nucleic Acids Res* **41**, W427-31 (2013).

4. Sievers, F. et al. Fast, scalable generation of high-quality protein multiple sequence alignments using Clustal Omega. *Mol Syst Biol* **7**, 539 (2011).

5. Page, R.D. TreeView: an application to display phylogenetic trees on personal computers. *Comput Appl Biosci* **12**, 357-8 (1996).
